# Improvement in Quality of Life, Pain and Function After Total and Unicompartmental Knee Replacement: A Secondary Analysis of 12-Month Post-Operative Outcomes

**DOI:** 10.1155/aort/6274196

**Published:** 2025-04-17

**Authors:** Deborah Snell, Jennifer Dunn, Gary Hooper

**Affiliations:** Department of Orthopedic Surgery and Musculoskeletal Medicine, University of Otago, P.O. Box 4345, Christchurch 8140, New Zealand

**Keywords:** patient-reported outcomes, quality of life, total knee replacement, unicompartmental knee replacement

## Abstract

**Objectives:** To investigate variables associated with improvement in quality of life (QOL) after primary knee replacement. QOL outcomes between individuals undergoing total knee replacement (TKR) and unicompartmental knee replacement (UKR) were compared.

**Materials and Methods:** Participants were adults (*n* = 497) undergoing TKR or UKR for osteoarthritis between January 2017 and October 2020 in a large publicly funded tertiary hospital in New Zealand. Participants completed patient-reported outcome measures of QOL, pain and function, preoperatively, 6 and 12 months postoperatively.

**Results:** QOL improved pre- to postoperatively for both TKR and UKR groups, and the main QOL gains for both groups were evident in the first 6 months after joint replacement. Notably, QOL did not differ between groups at any assessment point (*p* > 0.05). Improvement in QOL was more correlated with improved pain and function than with person factors such as demographics and comorbidity burden (*p* < 0.01).

**Conclusions:** This study adds to a growing literature showing that knee replacement contributes to substantial improvements in QOL outcomes. Future QOL outcome research in the knee replacement population should consider using more precise measures of function to better understand the impacts of these factors on QOL improvement.

## 1. Introduction

Outcomes following primary knee replacement are generally favourable for most individuals in terms of pain and function outcomes [1, 2]. Existing research on longer-term outcomes has tended to focus on these aspects and less on broader constructs such as quality of life (QOL) [3]. However, after achieving pain reduction, individuals may reassess their priorities and QOL and satisfaction outcomes may become more important in the longer term [4, 5]. Understanding factors associated with QOL improvement beyond the short-term post-operative period may help guide rehabilitation providers target outcomes relevant to the individual [6].

Studies have considered health-related QOL after knee replacement using patient-reported outcome measures that conflate pain and function with QOL, without considering broader aspects of an individual's environmental, social, psychological and general wellbeing [3]. The World Health Organisation (WHO) has considered QOL broadly to mean ‘an individual's perception of their life position, in the context of the culture and value systems in which they live and in relation to their goals, expectations, and concerns' [7, p. 1570]. This definition facilitated the development of a suite of generic QOL measures, including an 8-item short form of the WHOQOL-Bref, which has been validated for the joint replacement population [3]. This short form, called the EUROHIS-QOL 8-item index, incorporates items from each of the four WHOQOL-Bref domains (physical, psychological, social and environmental). The EUROHIS-QOL-8 is well tolerated [3] and offers an opportunity to explore factors associated with broader wellbeing outcomes after knee replacement.

Although factors affecting changes in QOL after knee replacement such as age, sex and comorbidities have been identified [4], these factors are inconsistent across studies and thus remain unclear [8]. In addition, the two main options for late-stage osteoarthritis of the knee are total knee replacement (TKR), or unicompartmental knee replacement (UKR) where only the damaged compartment of the knee is replaced [9]. There is disagreement among surgeons about the best surgical option and limited research evidence considering factors contributing to broader wellbeing and QOL outcomes among individuals undergoing TKR or UKR [10]. Identifying whether factors contributing to such wider outcomes differ among people undergoing TKR or UKR could help surgeons and rehabilitation providers tailor interventions to optimise outcomes.

### 1.1. Study Objectives

We aimed to investigate variables associated with improvement in QOL after primary knee replacement, evaluating QOL outcomes among individuals undergoing TKR and UKR. We hypothesised that those undergoing UKR would have lower levels of pain, higher function and QOL preoperatively, compared with those undergoing TKR, and both groups would show similar pre- to post-operative pain, function and QOL gains at 12 months, controlling for preoperative scores.

## 2. Materials and Methods

### 2.1. Design and Setting

This is a secondary analysis of data collected as part of a prospective observational study monitoring outcomes following joint replacement in Christchurch, New Zealand. We published a companion paper examining QOL outcomes among persons undergoing total hip replacement using similar methods [11]. Ethical and institutional approvals were received from New Zealand's National Health and Disability Ethics Committee (ref URA/09/01/EXP) and the University of Otago Human Ethics Committee (ref HD23/030).

### 2.2. Participants

The cohort consisted of a convenience sample of 497 individuals who had undergone TKR or UKR between January 2017 and October 2020. Surgical procedures took place in a large publicly funded tertiary hospital in Christchurch, New Zealand. Potential participants eligible for inclusion in the study were adults aged 18 or over who had undergone TKR or UKR and had agreed to participate in research following their progress over time. Participants provided written consent to participate in the research and completed outcome questionnaires by mail preoperatively, 6 months and 12 months postoperatively. Participants excluded from the study included those with significant comorbidities that impacted on their ability to complete study questionnaires, for example, admission to a dementia care facility and development of a serious comorbid condition such as late-stage cancer.

### 2.3. Data Collection and Measures

#### 2.3.1. Demographic and Clinical Variables

Demographic variables included age, gender and ethnicity. Surgical variables included the type of procedure (TKR, UKR). Comorbidities included (1) body mass index (BMI) and (2) the American Society of Anaesthesiologists (ASA) risk score or classification system [12]. The latter is a physical status classification system that enables clinicians to categorise a patient's physiological status and predict a patient's operative risk [13]. Classification scores are as follows: 1 (normally healthy), 2 (mild systemic disease), 3 (severe systematic disease, i.e., not incapacitating), 4 (incapacitating severe disease, i.e., a constant threat to life), 5 (moribund: survival not expected without surgery) and 6 (brain dead organ donor) [13]. As participants in the present study underwent elective procedures, ASA classifications of 5 and 6 were not applicable.

#### 2.3.2. QOL

An abbreviated version of WHOQOL-Bref questionnaire (EUROHIS-QOL 8-item index) [3, 14] has been validated for the joint replacement population [3]. It consists of eight items from the WHOQOL-Bref addressing the overall QOL, general health, energy, daily life activities, self-esteem, personal relationships and satisfaction with the home environment. Items from the EUROHIS-QOL 8-item index are summed to produce a single score (range 8–40) using the same response scale as the parent measure. Higher scores mean better QOL. For this study, all participants completed the EUROHIS-QOL 8-item index preoperatively, 6 and 12 months following primary TKR or UKR.

#### 2.3.3. Pain and Function

The Oxford Knee Score (OKS) [15] questionnaire is a specific measure of health status that focuses on the knee and contains 12 items scored on an ordinal scale (0–4) summed to a single score (range 0–48). For this study, higher scores indicated better functioning. Items assess pain and disability over the previous 4 weeks related to the joint that is being evaluated. The OKS has a simple scoring and summing system providing an overall score for assessing joint replacement outcome [16, 17]. OKS was collected preoperatively, 6 months and 12 months following primary TKR or UKR.

In addition to calculating a single summary scale score for the OKS, we also calculated pain and function subscale scores following Harris and colleagues [18]. Pain subscale scores were calculated from items 1, 8–12. Function subscale items were items 2–7. Subscale scores were transformed so that scores ranged from 0 (worst) to 100 (best).

### 2.4. Statistical Analyses

Data were analysed using SPSS version 28 [19]. Findings are reported following Strengthening the Reporting of Observational Studies in Epidemiology (STROBE) guidelines [20]. Demographic and clinical characteristics were summarised using descriptive statistics such as means, standard deviation, ranges and frequencies. Independent sample *t*-tests were used to evaluate differences between the TKR and UKR groups.

First, bivariate correlations (Pearson's *r*) were used to investigate whether change in pain and function preoperatively to 6 months and 12 months was associated with change in QOL. For each participant, preoperation scores were subtracted from scores at 6 months and 12 months post-operation. Changes in scores between 6 and 12 months were also calculated. The associations between these change scores were then investigated with Pearson's correlations. Because change scores are correlated with the scores at the first assessment (preoperation), a phenomenon known as regression to the mean [21], we also accounted for this potential effect. The residuals were saved from a regression model where the change score was the dependent variable and the preoperation scores were the independent variables. These residuals were analysed alongside the raw change scores. We then examined the EUROHIS-QOL item, pain and function correlations using Pearson's correlations. Finally, adjusted linear regression models further examined associations between demographic and clinical variables including all covariates in the model together, with change in QOL pre- to 12 months postoperation as the dependent variable.

Estimates were reported with two-sided 95% CIs. We used list-wise deletion, the default SPSS approach, to account for missing data. Data are reported as 95% confidence intervals (CIs). Effect sizes (ES) were calculated for significant results using Pearson *r* and Cohen's interpretation rules [22], as appropriate. Small, medium and large effect sizes were, respectively, defined as |0.1–0.3|, |0.3–0.5| and |0.5+| for Pearson's *r*; 0.2, 0.5 and 0.8 for Cohen's *d*; and 0.01, 0.09 and 0.25 for *R*^2^ [23]. A two-tailed *p* < 0.05 was used to evaluate statistical significance.

## 3. Results

### 3.1. Description of Study Sample

Data from 271 participants undergoing TKR and 226 participants undergoing UKR were analysed. The TKR sample was, on average, slightly older than the UKR sample (TKR mean 70.8 years, SD 9.0; UKR mean 69.5 years, SD 9.1). More men than women had a UKR (54%) compared with those who had a TKR (42.4%). In terms of comorbidities, participants in the TKR sample had higher BMIs on average (mean 32.2, SD 5.6) compared with those in the UKR sample (mean 30.6, SD 5.5) and higher levels of comorbidity as classified by the ASA score. Data on demographic and clinical characteristics are shown in Table 1.

Also shown in Table 1 are the differences between the two samples in pain, function and QOL at all three data collection points. Consistent with our predictions, TKR participants were more impaired across measures of pain and function at all time points with small to medium effect sizes evident. Contrary to our prediction, both groups endorsed similar levels of QOL at all three time points. Notably though, both TKR and UKR samples showed clinically significant improvements in pain, function and QOL preoperatively to 6 months postoperation but not between 6 and 12 months postoperation (Table 2).

### 3.2. QOL Item Associations With Pain and Function

When associations between individual EUROHIS-QOL items and pain subscale scores at each of the three assessment points were examined by procedure type, QOL items correlated positively with better pain outcomes at all time points (Table 3). The strongest correlations for TKR were between pain outcomes and overall perception of QOL (*r* = 0.4 to *r* = 0.5), satisfaction with health (*r* = 0.3 to *r* = 0.4) and satisfaction with the ability to perform activities of daily living (*r* = 0.4 to *r* = 0.4). The strongest correlations between pain outcomes and QOL items for UKR were with the overall perception of QOL (*r* = 0.5 to *r* = 0.6), satisfaction with health (*r* = 0.5), having enough energy (*r* = 0.4 to *r* = 0.5) and satisfaction with the ability to perform activities of daily living (*r* = 0.5 to *r* = 0.6).

When associations between individual EUROHIS-QOL items and function subscale scores at each of the three assessment points were examined by procedure type, similar patterns were evident (Table 4). While all QOL items correlated positively with better function outcomes, the strongest correlations for TKR were between function outcome and overall perception of QOL (*r* = 0.4 to *r* = 0.5), having enough energy (*r* = 0.3 to *r* = 0.5) and satisfaction with the ability to perform activities of daily living (*r* = 0.4 to *r* = 0.5). The strongest correlations between QOL items and function outcomes for UKR were with the overall perception of QOL (*r* = 0.5 to *r* = 0.6), satisfaction with health (*r* = 0.5) and satisfaction with the ability to perform activities of daily living (*r* = 0.5 to *r* = 0.7).

### 3.3. Variables Associated With Change in QOL

Table 5 shows that improvement in pain and function was correlated with improvement in QOL for both groups, but age and BMI were not. When demographic and clinical predictors of change in QOL pre- to 12 months postoperation were included in a multiple regression model, the overall model was significant for the TKR group (adjusted *R*^2^ = 0.14; *F* (6) = 5.13, *p* < 0.01). Only improvement in function contributed to variance in the QOL change. The model for UKR was not significant (adjusted *R*^2^ = 0.01; *F* (6) = 1.09, *p* = 0.37). These results are shown in Table 6.

## 4. Discussion

In this study we found that QOL improved from pre- to postoperation for both TKR and UKR groups. Notably, the main QOL gains for both groups were evident in the first 6 months after joint replacement. However, our hypotheses were only partially supported because at each assessment time point, while TKR participants demonstrated, on average, lower levels of function and higher pain, compared with UKR participants, both groups endorsed similar levels of QOL. Although effect sizes were small to medium, we also found that improvement in QOL was more correlated with improved pain and function than with person factors such as age or BMI, consistent with other studies [8, 24].

Inspection of individual QOL items showed that for both TKR and UKR groups, improved pain and function were particularly correlated with the improved overall perception of QOL, satisfaction with health and satisfaction with the ability to carry out activities of daily living. There were trends in the data towards change in function post-operatively being important for QOL among the TKR group. This finding is consistent with other studies evaluating QOL among people undergoing TKR. In a meta-analysis, Shan et al. [25] found that the factors that influenced QOL the most after TKR were the inability to go downstairs, inability to get around/do errands and pessimistic outlook on life. In another large study of the five factors contributing to 97% of variance in overall satisfaction following lower limb joint replacement, three were associated with pain and function (pain relief, preoperative and postoperative physical function) [26]. Further, in this study of 4709 participants, consistent with our findings, person factors such as age, gender and comorbidities did not impact on satisfaction. The quality of post-operative rehabilitation is also likely to contribute to improved pain, function and QOL, although optimal duration, frequency and intensity of rehabilitation remain unclear [27, 28]. We were not able to evaluate the influence of pre- and postoperative rehabilitation in the present study because we did not have data on access to rehabilitation.

One potential concern for our study is the choice of measure for evaluating change in function pre- to postoperatively. Previous studies have noted ceiling effects with the OKS function subscores [26, 29]. One study has suggested that while the OKS generally provides precise and discriminatory measurement in preoperative populations, individuals with higher OKS (greater than 40) postoperatively are measured with less precision [30]. For example, the OKS struggles to differentiate between an individual able to go back to gardening/shopping and one able to resume playing competitive tennis after knee replacement [30]. This may be particularly relevant to the UKR group where the mean total OKS at 6 and 12 months post-operatively in our study were close to the ceiling of the scale (6 months 41.5; 12 months 45.7). However, in general, it is well noted that people are living longer and want to stay active without knee pain and demands for TKR as well are increasing [31]. Return to sport rates for those undergoing UKR has been reported to be high (between 75% and 100%), also increasing among those undergoing TKR (39%–89%) [31]. In order to better measure the return to function and associations between function and QOL, while mitigating ceiling effects, future research should consider including more precise function measures such as the High Activity Arthroplasty Scale (HAAS) [32].

Other limitations include loss to follow-up. We did not use missing data imputation strategies, reflecting the pragmatic nature of the study, embedded within clinical practice. Further, data were collected from a sample recruited from a single region in NZ and findings may not be reflective of the wider NZ population of people with joint disease. Thus, there is potential for recruitment bias, especially regarding ethnicity. The Canterbury region of NZ has lower proportions of non-NZ European ethnicities than the general NZ population based on the 2018 census (https://www.stats.govt.nz/tools/2018-census-place-summaries/new-zealand#ethnicity-culture-and-identity). However, when we compared sample characteristics with those reported by a national joint registry (NZJR), other than ethnicity, mean age, gender mix, comorbidity rates, pain and function outcomes are similar to those described for TKR and UKR populations by the NZJR over 24 years of data collection [33]. Finally, there may be bias in our findings because both the EUROHIS-QOL and OKS include items evaluating function. However, wording within each of the measures differed. The QOL life measure focused more on satisfaction with function, and more nuanced difficulties with activities of daily living associated specifically with the knee were the focus of the OKS. This suggests that these measures are tapping different aspects of function. In addition, we did not have a control group available such as patients not undergoing knee replacement but receiving conservative treatment. Such a control group would have helped tease out impacts of surgical intervention on outcomes.

## 5. Conclusions

This study adds to a growing literature showing that knee replacement contributes to substantial improvements in QOL, pain and function outcomes. These relationships are likely to be inter-related and mutually reinforcing. Improvements in QOL can be seen by 6 months after both TKR and UKR. Future QOL outcome research in the knee replacement population should consider using more precise measures of function to better understand the impacts of these factors on QOL improvement.

## Figures and Tables

**Table 1:** Demographic and clinical characteristics of the study sample (*n* = 497).

Variable	TKR (*n* = 271)	UKR (*n* = 226)	Mean difference (95% confidence intervals)	Effect size^3^
	Mean (SD) or *N* (%)	Mean (SD) or *N* (%)		
Demographic characteristics				
Age ([years], mean [SD], range)^1^	70.8 (9.0), 26.0–90.9	69.5 (9.1), 47.8–92.1	1.3 (−0.3, 2.9)	0.2
Sex (male, *n* [%])	115 (42.4)	122 (54.0)	—	—
Ethnicity (*n* [%])				
New Zealand Māori	19 (7.0)	5 (< 0.1)	—	—
Non-New Zealand Māori	252 (93.0)	221 (97.8)		
Clinical characteristics				
Body mass index (*M* [SD], range)^1^	32.2 (5.6), 19.8–52.9	30.6 (5.5), 18.8–30.6	1.6 (0.4, 2.7)	0.3⁣^∗∗^
ASA (*n* [%])				
Category 1	14 (5.2)	34 (15.5)	—	—
Category 2	155 (58.3)	135 (61.4)		
Category 3	94 (35.3)	50 (22.7)		
Category 4	3 (1.1)	1 (0.5)		
Patient-reported outcome measures^1^				
Oxford total score (preoperation) (*M* [SD], range)	19.1 (7.3), 2.0–44.0	22.9 (7.7), 4.0–43.0	−3.8 (−5.1, −2.5)	0.5⁣^∗∗^
Oxford total score (6 M postoperation) (*M* [SD], range)	37.2 (7.7), 13.0–48.0	40.3 (7.2), 15.0–48.0	−3.1 (−4.7, −1.6)	0.4⁣^∗∗^
Oxford total score (12 M postoperation) (*M* [SD], range)	38.3 (15.8), 11.0–48.0	41.5 (7.0), 13.0–48.0	−2.7 (−4.3, −1.2)	0.4⁣^∗∗^
Oxford pain score^2^ (preoperation) (*M* [SD], range)	38.4 (15.8), 0.0–92.8	45.7 (16.6), 7.1–92.8	−7.3 (−10.2, −4.4)	0.5⁣^∗∗^
Oxford pain score^2^ (6 M postoperation) (*M* [SD], range)	79.8 (17.9), 25.0–100.0	85.1 (16.4), 28.6–100.0	−5.3 (−9.0, −1.6)	0.3⁣^∗^
Oxford pain score^2^ (12 M postoperation) (*M* [SD], range)	83.2 (18.0), 17.9–100.0	88.2 (15.2), 21.4–100.0	−5.0 (−8.5, −1.5)	0.3⁣^∗^
Oxford function score^2^ (preoperation) (*M* [SD], range)	42.2 (16.0), 10.0–90.0	51.0 (16.8), 10.0–90.0	−8.8 (−11.8, −5.9)	0.5⁣^∗∗^
Oxford function score^2^ (6 M postoperation) (*M* [SD], range)	74.9 (15.9), 25.0–100.0	83.2 (14.6), 35.0–100.0	−8.3 (−11.6, −5.0)	0.5⁣^∗∗^
Oxford function score^2^ (12 M postoperation) (*M* [SD], range)	78.0 (16.4), 25.0–100.0	85.2 (14.9), 30.0–100.0	−7.2 (−10.5, −4.0)	0.5⁣^∗∗^
EUROHIS-QOL (preoperation) (*M* [SD], range)	26.5 (5.3), 9.0–40.0	27.3 (5.8), 10.0–40.0	−0.8 (−1.7, 0.2)	0.1
EUROHIS-QOL (6 M postoperation) (*M* [SD], range)	32.0 (4.7), 15.0–40.0	32.4 (5.3), 13.0–40.0	−0.5 (−1.6, 0.6)	0.1
EUROHIS-QOL (12 M postoperation) (*M* [SD], range)	31.8 (5.5), 10.0–40.0	32.3 (5.9), 11.0–40.0	−0.5 (−1.7, 0.7)	0.1

*Note:* ASA = American Society of Anaesthesiologists (ASA) risk score.

Abbreviations: TKR = total knee replacement, UKR = unicompartmental knee replacement.

^1^Independent sample *t*-tests.

^2^Oxford pain and function subscale scores are transformed scores.

^3^Effect sizes are absolute.

⁣^∗^*p* < 0.05.

⁣^∗∗^*p* < 0.01.

**Table 2:** Change in patient-reported outcomes over time (*n* = 497).

Variable	TKR (*n* = 271)	UKR (*n* = 226)	Mean difference (95% confidence intervals)	Effect size^2^
	Mean (SD)	Mean (SD)		
Change in Oxford total score pre-6 M postsurgery	17.9 (8.9)	17.7 (8.7)	0.2 (−1.7, 2.1)	0.0
Change in Oxford total score pre-12 M postsurgery	19.0 (9.3)	18.1 (8.0)	0.9 (−0.9, 2.7)	0.0
Change in Oxford total score 6 M–12 M postsurgery	1.1 (6.4)	1.1 (5.3)	0.0 (−1.3, 1.4)	0.1
Change in Oxford pain score pre-6 M postsurgery^1^	41.7 (21.1)	40.4 (20.0)	1.3 (−3.2, 5.8)	0.1
Change in Oxford pain score pre-12 M postsurgery^1^	43.3 (22.0)	41.3 (17.9)	2.0 (−2.2. 6.2)	0.1
Change in Oxford pain score 6 M–12 M postsurgery^1^	2.7 (14.9)	2.1 (11.8)	0.7 (−2.6, 3.9)	0.1
Change in Oxford function score pre–6 M postsurgery^1^	32.0 (17.9)	33.4 (18.0)	−1.4 (−5.3, 2.5)	0.1
Change in Oxford function score pre–12 M postsurgery^1^	34.8 (19.3)	33.7 (17.7)	1.1 (−2.8, 5.0)	0.1
Change in Oxford function score 6 M–12 M postsurgery^1^	1.7 (13.9)	1.6 (11.2)	0.2 (−2.9, 3.2)	0.0
Change in QOL pre–6 M postsurgery	5.1 (4.7)	5.1 (4.6)	0.0 (−1.0, 1.0)	0.0
Change in QOL pre–12 M postsurgery	5.1 (5.2)	4.8 (4.8)	0.4 (−0.7, 1.5)	0.1
Change in QOL 6 M–12 M postsurgery	−0.4 (3.7)	0.0 (3.4)	−0.4 (−1.3, 0.4)	0.1

Abbreviations: TKR = total knee replacement, UKR = unicompartmental knee replacement.

^1^Oxford pain and function subscale scores are transformed scores.

^2^Effect sizes are absolute; QOL measured with EUROHIS-QOL 8-item index.

⁣^∗^*p* < 0.05.

⁣^∗∗^*p* < 0.01.

**Table 3:** EUROHIS-QOL item correlations with pain at surgery preoperation, 6 and 12 months postoperation.

EUROHIS-QOL item	Pain preoperation		Pain 6 months postoperation		Pain 12 months postoperation	
	Pearson's *R*		Pearson's *R*		Pearson's *R*	
	UKR	TKR	UKR	TKR	UKR	TKR
(1) Overall quality of life^a^						
Preoperation	0.52⁣^∗∗^	0.44⁣^∗∗^				
6 months postoperation			0.62⁣^∗∗^	0.52⁣^∗∗^		
12 months postoperation					0.48⁣^∗∗^	0.44⁣^∗∗^
(2) Satisfaction with health^b^						
Preoperation	0.45⁣^∗∗^	0.24⁣^∗∗^				
6 months postoperation			0.46⁣^∗∗^	0.31⁣^∗∗^		
12 months postoperation					0.45⁣^∗∗^	0.30⁣^∗∗^
(3) Enough energy for everyday life^c^						
Preoperation	0.38⁣^∗∗^	0.31⁣^∗∗^				
6 months postoperation			0.48⁣^∗∗^	0.41⁣^∗∗^		
12 months postoperation					0.43⁣^∗∗^	0.37⁣^∗∗^
(4) Enough money to meet needs^c^						
Preoperation	0.25⁣^∗∗^	0.18⁣^∗∗^				
6 months postoperation			0.21⁣^∗∗^	0.21⁣^∗∗^		
12 months postoperation					0.30⁣^∗∗^	0.26⁣^∗∗^
(5) Satisfaction with the ability to perform daily living activities^b^						
Preoperation	0.55⁣^∗∗^	0.40⁣^∗∗^				
6 months postoperation			0.60⁣^∗∗^	0.41⁣^∗∗^		
12 months postoperation					0.52⁣^∗∗^	0.43⁣^∗∗^
(6) Satisfaction with self^b^						
Preoperation	0.41⁣^∗∗^	0.29⁣^∗∗^				
6 months postoperation			0.51⁣^∗∗^	0.35⁣^∗∗^		
12 months postoperation					0.47⁣^∗∗^	0.32⁣^∗∗^
(7) Satisfaction with personal relationships^b^						
Preoperation	0.22⁣^∗∗^	0.06				
6 months postoperation			0.32⁣^∗∗^	0.16⁣^∗^		
12 months postoperation					0.41⁣^∗∗^	0.25⁣^∗∗^
(8) Satisfaction with the conditions of living place^b^						
Preoperation	0.19⁣^∗∗^	0.07				
6 months postoperation			0.32⁣^∗∗^	0.19⁣^∗^		
12 months postoperation					0.36⁣^∗∗^	0.24⁣^∗∗^

*Note:* Higher pain scores = better outcome.

^a^Likert options: 1 = very poor, 2 = poor, 3 = neither poor nor good, 4 = good, 5 = very good.

^b^Likert options: 1 = very dissatisfied, 2 = dissatisfied, 3 = neither satisfied nor dissatisfied, 4 = satisfied, 5 = very satisfied.

^c^Likert options: 1 = not at all, 2 = a little, 3 = a moderate amount, 4 = very much, 5 = extremely.

⁣^∗^*p* < 0.05.

⁣^∗∗^*p* < 0.01.

**Table 4:** EUROHIS-QOL item correlations with function at surgery pre-operation, 6 and 12 months postoperation.

EUROHIS-QOL item	Function preoperation		Function 6 months postoperation		Function 12 months postoperation	
	Pearson's *R*		Pearson's *R*		Pearson's *R*	
	UKR	TKR	UKR	TKR	UKR	TKR
(1) Overall quality of life^a^						
Preoperation	0.53⁣^∗∗^	0.42⁣^∗∗^				
6 months postoperation			0.61⁣^∗∗^	0.53⁣^∗∗^		
12 months postoperation					0.51⁣^∗∗^	0.45⁣^∗∗^
(2) Satisfaction with health^b^						
Preoperation	0.46⁣^∗∗^	0.24⁣^∗∗^				
6 months postoperation			0.49⁣^∗∗^	0.40⁣^∗∗^		
12 months postoperation					0.48⁣^∗∗^	0.34⁣^∗∗^
(3) Enough energy for everyday life^c^						
Preoperation	0.37⁣^∗∗^	0.33⁣^∗∗^				
6 months postoperation			0.49⁣^∗∗^	0.40⁣^∗∗^		
12 months postoperation					0.43⁣^∗∗^	0.37⁣^∗∗^
(4) Enough money to meet needs^c^						
Preoperation	0.32⁣^∗∗^	0.20⁣^∗∗^				
6 months postoperation			0.33⁣^∗∗^	0.29⁣^∗∗^		
12 months postoperation					0.33⁣^∗∗^	0.21⁣^∗∗^
(5) Satisfaction with the ability to perform daily living activities^b^						
Preoperation	0.51⁣^∗∗^	0.41⁣^∗∗^				
6 months postoperation			0.69⁣^∗∗^	0.49⁣^∗∗^		
12 months postoperation					0.57⁣^∗∗^	0.48⁣^∗∗^
(6) Satisfaction with self^b^						
Preoperation	0.41⁣^∗∗^	0.33⁣^∗∗^				
6 months postoperation			0.54⁣^∗∗^	0.41⁣^∗∗^		
12 months postoperation					0.50⁣^∗∗^	0.34
(7) Satisfaction with personal relationships^b^						
Preoperation	0.19⁣^∗∗^	0.16⁣^∗∗^				
6 months postoperation			0.31⁣^∗∗^	0.17⁣^∗^		
12 months postoperation					0.49⁣^∗∗^	0.24⁣^∗∗^
(8) Satisfaction with the conditions of living place^b^						
Preoperation	0.19⁣^∗∗^	0.13⁣^∗^				
6 months postoperation			0.35⁣^∗∗^	0.26⁣^∗∗^		
12 months postoperation					0.42⁣^∗∗^	0.23⁣^∗∗^

*Note:* Higher function scores = better outcome.

^a^Likert options: 1 = very poor, 2 = poor, 3 = neither poor nor good, 4 = good, 5 = very good.

^b^Likert options: 1 = very dissatisfied, 2 = dissatisfied, 3 = neither satisfied nor dissatisfied, 4 = satisfied, 5 = very satisfied.

^c^Likert options: 1 = not at all, 2 = a little, 3 = a moderate amount, 4 = very much, 5 = extremely.

⁣^∗^*p* < 0.05.

⁣^∗∗^*p* < 0.01.

**Table 5:** Correlations between the change in quality of life from preoperation to 12 months postoperation and demographic and clinical variables among people undergoing total and unicompartmental knee replacement.

Variable	QOL preop-12 M postop change scores (raw)		QOL preop-12 M postop change scores (residual)	
	Pearson's *r*	*p*-value	Pearson's *r*	*p*-value
Total knee replacement (*n* = 271)				
Age	−0.08	0.28	0.05	0.53
BMI	0.11	0.18	−0.03	0.68
Change in Oxford pain score pre-12 M postsurgery^1^	0.31	< 0.01	0.31	< 0.01
Change in Oxford function score pre-12 M postsurgery^1^	0.38	< 0.01	0.35	< 0.01
Unicompartmental knee replacement (*n* = 226)				
Age	0.02	0.80	0.06	0.48
BMI	0.12	0.22	−0.02	0.82
Change in Oxford pain score pre-12 M postsurgery^1^	0.32	< 0.01	0.32	< 0.01
Change in Oxford function score pre-12 M postsurgery^1^	0.22	0.01	0.20	0.01

*Note:* QOL = quality of life measured with the EUROHIS-QOL 8-item index.

Abbreviation: BMI = body mass index.

^1^Oxford pain and function subscale scores are transformed scores.

**Table 6:** Multivariate linear regression models evaluating associations with the change in quality of life after knee replacement (*n* = 497).

Model	Adjusted *R*^2^	*B*	95% CI *B*	
			Lower	Upper
Total knee replacement	**0.14 **⁣^∗^			
Block 1				
Gender (female)		−1.24	−2.92	0.44
Age		−0.03	−0.13	0.06
Block 2				
Body mass index		0.07	−0.07	0.22
ASA		2.06	−2.28	6.40
Block 3				
Change in Oxford pain score (pre-12 months postsurgery)		0.03	−0.03	0.08
Block 4				
Change in Oxford function score (pre-12 months postsurgery)		**0.08 **⁣^∗∗^	0.02	0.14
Unicompartmental knee replacement	0.07			
Block 1				
Gender (female)		1.15	−0.98	3.28
Age		−0.05	−0.20	0.10
Block 2				
Body mass index		0.04	−0.17	0.26
ASA		−0.22	−3.62	3.18
Block 3				
Change in Oxford pain score (pre-12 months postsurgery)		0.06	−0.01	0.13
Block 4				
Change in Oxford function score (pre-12 months postsurgery)		−0.01	−0.09	0.06

*Note:* Dependent variable = Change in QOL as measured with the EUROHIS-QOL 8-item index pre- to 12-month postoperation, ASA = American Society of Anaesthesiologists risk score. Bolded values are significant.

⁣^∗^*p* < 0.05.

⁣^∗∗^*p* < 0.01.

## Data Availability

Data are available on request.
